# Association between patient characteristics and recommendations by medical on-call service 116117 in Germany: a cross sectional observational study

**DOI:** 10.1186/s12911-025-02970-4

**Published:** 2025-03-31

**Authors:** Heike Hansen, Agata Menzel, Jan Hendrik Oltrogge-Abiry, Dagmar Lühmann, Martin Scherer, Ingmar Schäfer

**Affiliations:** Department of Primary Medical Care, University Medical Center Hamburg-Eppendorf, Martinistr. 52, 20246 Hamburg, Germany

**Keywords:** Patient characteristics, Recommendations by medical on-call service, Specific setting recommendations, Cross sectional observational study

## Abstract

**Background:**

Use of emergency departments has increased in recent years. Different efforts address this problem, eg, medical on-call services. The basis of the DEMAND intervention is computer-assisted initial telephone assessment implemented at regional associations of statutory health insurance physicians in Germany. In this intervention, recommendations for healthcare settings were given over the telephone by medical staff. Recommendations were provided using the software SmED which calculates neural networks. This study aimed to analyse if patient characteristics are associated with the output of the intervention, ie, specific setting recommendations.

**Methods:**

Between January 2020 and March 2021, patients aged 18 years and older of the DEMAND intervention from eight intervention sites received a standardised postal survey. Recommended and used settings, and data on sociodemography, health status at the time of the emergency call, past health service use, and health literacy were collected by self-report. Multilevel, multivariable logistic regression models adjusted for random effects at the level of regions and months of observation within regions were conducted.

**Results:**

Of 9473 contacted individuals, 1756 (18.5 %) participated in the survey. Median age was 66 years, 59.0% were women and 30.2% living alone. The most frequently recommended service was emergency home visits (40.1%). Recommendations for this setting were associated with worse self-rated health (odds ratio 0.67, 95% confidence interval: 0.55/0.81, *p* < 0.001). Telephone counselling was associated with lower age (0.71, 0.59/0.85, *p* < 0.001), lower subjective treatment urgency (0.65, 0.51/0.82, *p* < 0.001) and health problems not classified as symptoms and complaints (0.41, 0.25/0.68, *p* = 0.001) or infections (0.22, 0.09/0.57, *p* = 0.002). Emergency departments were associated with better self-rated health (1.37, 1.11/1.70, *p* = 0.003) and health problems classified as injuries (3.12, 1.67/5.83, *p* < 0.001). Rescue service were associated with higher age (1.44, 1.15/1.81, *p* = 0.002) and higher subjective treatment urgency (2.51, 1.83/3.43, *p* < 0.001). General practices were associated with lower subjective treatment urgency (0.58, 0.44/0.76, *p* < 0.001) and health problems not classified as injuries (0.26, 0.10/0.68, *p* = 0.006). Emergency practices were associated with lower age (0.60, 0.48/0.74, *p* < 0.001), and specialist practices were associated with health problems classified as symptoms or complaints (3.75, 1.49/9.45, *p* = 0.005).

**Conclusions:**

Most associations between patient characteristics and recommendations were comprehensible and in line with the aim of the intervention. However, it should be clarified why patients with better self-rated health were more likely to receive recommendations for emergency departments.

## Background

In Germany and many other countries, the use of emergency departments has increased significantly in recent years, particularly by patients with less urgent healthcare needs [1–4]. A German study identified that more than half of the patients visiting an emergency department had low subjective treatment urgency. In addition to the urgency of the health problem, the decision to visit an emergency department can also be affected by perceived structural circumstances, eg, regarding availability of services, and individual patient preferences, eg, assumptions about the quality of diagnostic and treatment facilities [5].

There are different efforts for managing the increased emergency department utilisation, eg, medical on-call services that utilise computer-assisted telephone triage systems. These systems are designed to coordinate patient pathways within the healthcare system after evaluating the symptoms over phone [6]. However, evidence in this topic is limited and inconsistent. Therefore, further evaluations are required [7].

Few studies examined the associations between patient characteristics and recommendations by medical on-call services. Dale et al. reported an association of older age and higher rates of referral for home visits for a nursed-led out-of-hours telephone triage and advice service in general practice [8]. Another study found that older adults and patients with diabetes mellitus, dementia, or previous cerebral infarction were at risk of being inappropriately triaged [9].

In the German Health Care System, available healthcare settings for emergencies are rescue services (emergency treatment on site and transport to the hospital), emergency departments (ambulatory treatment at hospital), emergency home visits (visit by outpatient physicians), emergency practices (outpatient treatment outside office hours), telephone counselling (advice by physicians), specialist care (outpatient specialist care during office hours) and general practice (primary care during office hours). Patients are free to decide which emergency service they use. Outpatient emergency services are managed by the regional Associations of Statutory Health Insurance Physicians (ASHIPs) [10].

The DEMAND (“implementation of a standardized initial assessment as the basis of DEMAND management in outpatient emergency care”) intervention is one of various strategies to improve patient allocation in out-of-hours care [10–13]. Since January 2020, DEMAND is implemented at the medical on-call services (“116117”) of eight ASHIPs located in the German federal states of Bavaria, Brandenburg, Bremen, Hesse, North Rhine-Westphalia (represented by two ASHIPs in the regions North Rhine and Westphalia-Lippe), Schleswig-Holstein and Thuringia.

Core element of this intervention is the software SmED (“Strukturierte medizinische Ersteinschätzung in Deutschland”). SmED provides recommendations on treatment urgency and appropriate care settings according to the current state of evidence by analyzing the patient-reported medical history using neural networks. The SmED software had been developed on the basis of the established software SMASS (“Swiss Medical Assessment System”) [14, 15]. In the DEMAND intervention, medical staff conducted computer-assisted structured initial assessments using SmED and gave recommendations for healthcare settings appropriate to the patients’ health problems.

The accompanying research to the DEMAND intervention monitored the implementation process and analysed associations between views on SmED, use of SmED and patient characteristics [16]. In general, initial experiences of healthcare workers with the use of SmED were positive [17]. Other studies described patient characteristics and consultation reasons in the initial assessments [18] and reported satisfying rates of compliance with the generated recommendations and patient satisfaction with the intervention [10]. Unpublished analyses based on health insurance data described a negative association between SmED implementation and emergency department utilisation in most regions and no increase in the documented mortality rates due to SmED use [19]. However, validity of the recommendations has not been investigated, yet.

Therefore, our study aimed to analyse if individual patient characteristics are associated with the output of the DEMAND intervention, ie, with specific setting recommendations.

## Methods

Patients participating in the DEMAND intervention received a standardised postal survey. Intervention and survey were described in detail in another paper [10]. In short, at the medical on call service 116117, trained medical staff conducted computer-assisted structured initial assessment with the patient via telephone and gave recommendations of suitable treatment settings after evaluation of patients’ specific situation. The telephone service was available 24 h at seven days a week. Each initial assessment lasted for approximately 2.5 min [20] and consisted of two steps. First, using SmED, immediate threat to life was excluded. Second, based on symptom and health data, treatment urgency was calculated and suitable healthcare settings were proposed by SmED. These recommendations were then discussed between the medical staff and patients considering availability of services (eg, by region and time of day) and the specific situation of the patient (including additional health complaints and available transport). Rescue service, home visits and telephone counselling were organised by the medical staff, the other settings were utilised by the patients [10].

The DEMAND intervention was examined in the telephone services of the eight ASHIPs described above. An English translation of the German questionnaire was published in the supplements of the paper by Schäfer et al. 2023 [10], which described compliance and patient satisfaction with the intervention. Both factors depended on the recommended settings. For example, 68.4% of the patients followed the recommendation “GP practice” and 75.9% of these patients were satisfied with the intervention. In contrast, 80.4% followed the recommendation “emergency home visit” and the satisfaction rate among these patients was 84.6% [10]. DEMAND has also been implemented in selected hospitals, which has been analysed in another study [21].

### Selection of participants and data collection

Due to a strict approval process for transferring personally identifying information by the German federal governments, the beginning of data collection was different between regions. The observation time in North Rhine-Westphalia and Bremen was between January and December 2020, in Brandenburg between April 2020 and March 2021, in Bavaria between June 2020 and February 2021, in Hesse between July 2020 and March 2021, in Thuringia between October 2020 and March 2021 and in Schleswig-Holstein between January and March 2021 [10].

After each finished month of the observation time the respective ASHIP produced list of patients aged 18 years or older who took part in the intervention. Patients were excluded if no valid postal address was reported. The study centre received the contact data by electronic data transmission. A total of 10,000 patients from 63 lists were randomly selected stratified by intervention site and month of observation. Questionnaire and patient information were sent by mail to the selected patients. In 43 lists, patients had a time lag between 4 and 47 days, in 14 lists between 18 and 58 days, and in 6 lists between 34 and 72 days between intervention and survey.

Criteria for excluding patients retrospectively were: incorrect postal address, patients reported that they did not use the telephone service of the respective ASHIP, another household member reported that the patients were not able to fill out the questionnaire (eg, due to functional limitations) or patients had died. The patients gave their informed consent to study participation by sending back the completed questionnaires in anonymous form to the study centre. The study was approved by the Ethics Committee of the Hamburg Medical Association on 08 April 2019 (approval no. PV6035) [10].

### Measurements

Endpoint of our analyses were recommended settings of emergency care, which were collected by patient self-report. Multiple answers were possible. Patients’ satisfaction with the computer-assisted initial assessment and the used setting were evaluated by rating their agreement to eight statements on a four-point Likert scale [10].

Data on sociodemography, health status at the time of the emergency call, past health service use, and health literacy were collected. Sociodemographic data involved age, sex, living arrangement, educational level of the patients, and country of birth of the study participants and their parents. The educational level was coded according to the CASMIN classification [22] in three hierarchical categories. A visual analogue scale ranging from 0 (indicating the worst) to 100 (indicating the best imaginable health status at the day of the initial assessment) was used for assessing self-rated health.

Patients rated their subjective treatment urgency on a numerical rating scale ranging from 0 (indicating no urgent need for treatment) to 10 (indicating very urgent need for treatment and/or life threatening condition). To screen for depressed mood and anhedonia in the two weeks before initial assessment PHQ-2 (Patient Health Questionnaire) [23] was used. It reveals a summary score ranging between 0 (indicating no symptoms) and 8 (indicating both symptoms occurring almost every day). Patients reported their consultations reasons by open questions. JHO and AM coded these information retrospectively in the International Classification of Primary Care, Second Revision (ICPC-2) [24], which facilitates grouping by organ system and diagnosis type (eg, “symptoms/complaints”, or “infections”). In cases of multiple possible codings of the available information, the raters were instructed to choose the most specific codes, eg, specific diagnoses were preferred over unspecific symptoms. Health problems were considered by diagnosis groups and organ systems with a prevalence ≥ 5%.

The past health service use in the three months before the initial assessment (general practices, specialist practices, hospitals, and emergency services) was also collected by patient report. To collect health literacy we used the European Health Literacy Questionnaire HLS-EU-Q16 rated on a four-point Likert scale. For the analyses, we dichotomised the data. The summary score for health literacy was assigned to three hierarchical categories: inadequate (0–8 points), problematic (9–12 points) and sufficient (13–16 points) [25, 26].

### Statistical analyses

The study population was characterised by descriptive statistics. For analysing the association between recommendations, and patient characteristics we conducted multilevel, multivariable logistic regression models. In order to account for potentially reduced variance due to clustering, we adjusted the models for random effects at the level of regions and months of observation within regions.

Independent variables of each model were age (continuous), sex (categorical), living arrangement (categorical), education (categorical), country of birth (categorical), subjective treatment urgency (continuous), self-rated health (continuous), depressiveness (continuous), organ system (categorical) and diagnosis type (categorical) of the health problem, past health service use (continuous), and health literacy (categorical). The respective setting recommendations were defined as dependent variables, which resulted in seven statistical tests.

As the study was conducted without pre-planned hypotheses [27] and in order to obtain a conservative estimate of intervention effects, we performed Bonferroni-adjustment for these analyses and defined an Alpha-level of *p* ≤ 0.007 as statistically significant. All statistical analyses were conducted using Stata 15.1. and based on the available data.

## Results

### Characteristics of the study population

The recruitment process and the study population had been described elsewhere in detail [10]. Briefly summarized, 408,781 patients were checked for eligibility. 67,355 patients had to be excluded. Thereof, 46,140 had an age less than 18 years or unknown, 14,998 were double entries, and for 8970 contact information was missing. Out of the remaining 341,426 eligible patients, we randomly selected and contacted 10,000 patients. Due to exclusion criteria or death or functional limitations 527 patients had to be retrospectively excluded. In the end, 1756 patients participated in the survey (18.5 % response rate).

The median age of the patients was 66 years (interquartile range: 50–79), 59.0% were women and 30.2% were living alone. 23.0% of the patients had a tertiary educational level, 43.4% had a secondary educational level and 33.7% had uncompleted education or primary educational level. The majority of the patients was born in Germany (85.9%), and few patients were born abroad (8.2%) or had at least one parent born abroad (5.9%). The median subjective treatment urgency was 7 (interquartile range: 5–8). The median of self-rated health on the day of the intervention was 40 (interquartile range: 24–60) on a scale ranging 0–100. 41.4% of the patients had a sufficient health literacy, 36.2% problematic and 22.4% inadequate.

The organ system with the highest prevalence was “general and unspecified”, which included symptoms and complaints like “fever” (10.2%), “feeling ill” (5.2%), and “chest pain, not otherwise specified” (4.3%). 68.7% of the patients reported symptoms or complaints, 6.9% injuries, 5.5% infections and 19.6% other diagnoses as health problems. In the last six months before participating in the intervention, 67.8% had visited a general practice, 45.3% a specialist practice, 21.7% utilised inpatient care, and 18.1% emergency care.

Recommended and used settings after computer-assisted structured initial assessment are presented in Table 1. A total of 40.1% of the patients received a recommendation for getting emergency home visits, followed by telephone counselling (20.4%), emergency department (20.2%), rescue service (17.6%), general practices (13.1%), emergency practices (12.3%) and specialist practices (4.5%).

**Table 1:** Proportions of recommended and used settings* after computer-assisted structured initial assessment

Setting	Recommended (n = 1684)	Used (n = 1635)
Emergency home visit	40.1% (n = 676)	35.3% (n = 577)
Telephone counselling	20.4% (n = 343)	17.4% (n = 232)
Emergency department	20.2% (n = 340)	23.2% (n = 379)
Rescue service	17.6% (n = 296)	20.7% (n = 338)
General practice	13.1% (n = 221)	14.2% (n = 232)
Emergency practice	12.3% (n = 207)	9.7% (n = 159)
Specialist practice	4.4% (n = 75)	6.2% (n = 102)

*Data are not mutually exclusive

### Differences between recommended settings in patient characteristics

The descriptive distributions of sociodemographic and health-related data by recommended settings are shown in Table 2a, b. Compared to the total population, patients receiving recommendations for rescue service were older (71 vs. 66 years), had less often attained tertiary education (14.5% vs. 23.0%), worse self-rated health (30 vs. 40 points) (cf. Table 2a), more often health problems in the cardiovascular system (29.6% vs. 15.7%), more often other diagnoses than infections or injuries (31.9% vs. 19.6%), more often visited general practices (76.2% vs. 67.8%), hospitals (33.6% vs. 21.7%) and emergency care (25.6% vs. 18.1%) in the last three months, and had more often inadequate health literacy (28.4% vs. 22.4%) (cf. Table 2b).

**Table 2a:** Descriptive data of the distributions of sociodemographic and health-related data by recommended settings

	Total (n = 1684)	Rescue service (n = 296)	Emergency home visit (n = 676)	Emergency department (n = 340)	Emergency practice (n = 207)	Specialist practice (n = 75)	General practice (n = 221)	Telephone counselling (n = 343)
Age: median [interquartile range]	66 [50–79]	71 [61–81]	67 [53–80]	68 [52–79]	55 [37–68]	59 [39–71]	64 [47–77]	61 [37–73]
	(n = 1660)	(n = 289)	(n = 667)	(n = 334)	(n = 205)	(n = 75)	(n = 219)	(n = 342)
Sex:								
–Women	59.0%	58.4%	59.9%	58.5%	56.8%	53.3%	59.6%	65.5%
–Men	40.8%	41.6%	40.0%	41.3%	42.7%	46.7%	40.5%	34.2%
–Non-binary	0.2%	-	0.2%	0.3%	0.5%	-	-	0.3%
	(n = 1670)	(n = 291)	(n = 670)	(n = 337)	(n = 206)	(n = 75)	(n = 220)	(n = 342)
Living arrangement:								
–Living alone	30.2%	32.3%	32.3%	32.1%	23.0%	31.1%	32.7%	30.3%
–Living together with others	69.8%	67.7%	67.7%	67.9%	77.0%	68.9%	67.3%	69.7%
	(n = 1652)	(n = 288)	(n = 660)	(n = 333)	(n = 204)	(n = 74)	(n = 217)	(n = 337)
Education (pursuant to CASMIN):								
–Uncompleted, general elementary or basic vocational	33.7%	41.7%	36.2%	35.7%	20.7%	21.9%	35.1%	24.4%
–Secondary school certificate or “A” level equivalent	43.4%	43.8%	44.0%	43.1%	51.7%	42.5%	43.0%	50.0%
–Higher or lower tertiary	23.0%	14.5%	19.8%	21.2%	27.6%	35.6%	22.0%	25.6%
	(n = 1631)	(n = 283)	(n = 652)	(n = 325)	(n = 203)	(n = 73)	(n = 214)	(n = 336)
Country of birth:								
–Patient and both parents in Germany	85.9%	84.1%	85.3%	87.8%	87.4%	84.9%	83.6%	82.7%
–Patient in Germany and at least one parent abroad	5.9%	5.5%	5.4%	5.4%	6.8%	8.2%	8.2%	7.9%
–Patient abroad	8.2%	10.3%	9.3%	6.9%	5.8%	6.9%	8.2%	9.4%
	(n = 1660)	(n = 290)	(n = 665)	(n = 336)	(n = 207)	(n = 73)	(n = 219)	(n = 340)
Subjective treatment urgency (numerical rating scale: median [interquartile range])	7 [5–8]	8 [7–9]	7 [6–8]	7 [6–8]	7 [5–8]	6 [4–8]	6 [5–8]	6 [5–8]
	(n = 1580)	(n = 272)	(n = 635)	(n = 324)	(n = 203)	(n = 69)	(n = 204)	(n = 326)
Self-rated health (EQ-5D visual analogue scale: median [interquartile range])	40 [24–60]	30 [20–50]	34 [20–50]	40 [30–60]	50 [30–70]	50 [29–70]	50 [30–60]	40 [30–60]
	(n = 1640)	(n = 289)	(n = 660)	(n = 331)	(n = 204)	(n = 73)	(n = 214)	(n = 337)
Depressiveness (pursuant to PHQ-2: median [interquartile range])	1 [0–2]	2 [0–3]	1 [0–3]	1 [0–2]	0 [0–2]	1 [0–3]	1 [0–2]	1 [0–2]
	(n = 1493)	(n = 255)	(n = 598)	(n = 289)	(n = 193)	(n = 68)	(n = 201)	(n = 321)

**Table 2b:** Descriptive data of the distributions of sociodemographic and health-related data by recommended settings (continued)

	Total (n = 1684)	Rescue service (n = 296)	Emergency home visit (n = 676)	Emergency department (n = 340)	Emergency practice (n = 207)	Specialist practice (n = 75)	General practice (n = 221)	Telephone counselling (n = 343)
Health problem: organ system (pursuant to ICPC-2):								
–General and unspecified disorders	23.3%	27.4%	24.6%	19.8%	18.4%	16.2%	25.4%	24.1%
–Musculoskeletal system	21.9%	19.3%	26.0%	22.2%	25.0%	28.4%	22.0%	14.8%
–Digestive system	18.4%	19.6%	19.3%	23.2%	11.7%	14.9%	16.6%	18.1%
–Cardiovascular system	15.7%	29.6%	12.9%	17.6%	12.2%	10.8%	11.2%	17.5%
–Respiratory system	13.5%	13.3%	14.0%	11.1%	18.4%	9.5%	14.2%	13.0%
–Neurological system	9.1%	11.1%	10.4%	7.1%	7.7%	4.1%	8.3%	10.8%
–Urological system	6.1%	4.8%	7.6%	8.6%	7.1%	2.7%	3.4%	4.8%
	(n = 1595)	(n = 270)	(n = 643)	(n = 324)	(n = 196)	(n = 74)	(n = 205)	(n = 332)
Health problem: diagnosis type (pursuant to ICPC-2):								
–Symptoms and complaints	68.7%	65.6%	71.7%	71.6%	66.3%	73.0%	62.9%	62.7%
–Infections	5.5%	3.7%	6.7%	5.3%	9.2%	2.7%	4.4%	3.0%
–Injuries	6.9%	7.4%	5.0%	9.6%	8.7%	8.1%	4.9%	6.0%
–Other diagnoses	19.6%	31.9%	19.8%	19.8%	14.8%	18.9%	20.0%	19.0%
	(n = 1595)	(n = 270)	(n = 643)	(n = 324)	(n = 196)	(n = 74)	(n = 205)	(n = 332)
Past health service use in the last three months:								
–General practices	67.8%	76.2%	69.7%	70.7%	58.1%	67.6%	67.2%	63.9%
–Specialist practices	45.3%	44.0%	46.4%	48.5%	44.9%	59.2%	43.5%	46.4%
–Hospitals	21.7%	33.6%	23.2%	28.4%	17.6%	28.2%	19.8%	21.1%
–Emergency care	18.1%	25.6%	22.3%	24.1%	19.5%	18.3%	12.6%	13.3%
	(n = 1605)	(n = 277)	(n = 647)	(n = 324)	(n = 205)	(n = 71)	(n = 207)	(n = 332)
Health literacy (pursuant to HLS-Q16-EU):								
–Inadequate (0–8 points)	22.4%	28.4%	25.4%	25.3%	15.4%	11.6%	20.3%	20.9%
–Problematic (9–12 points)	36.2%	34.7%	35.8%	36.7%	37.4%	42.0%	36.6%	39.1%
–Sufficient (13–16 points)	41.4%	36.9%	38.8%	38.0%	47.2%	46.4%	43.1%	40.0%
	(n = 1551)	(n = 271)	(n = 623)	(n = 308)	(n = 195)	(n = 69)	(n = 202)	(n = 330)

Patients with recommendation for the emergency department had more often visited hospitals (28.4% vs. 21.7%) and emergency care (24.1% vs. 18.1%) in the last three months (cf. Table 2b). Patients recommended to visit emergency practices were younger (55 vs. 66 years), less often living alone (23.0% vs. 30.2%), had better self-rated health (50 vs. 40 points) (cf. Table 2a), less often health problems in the digestive system (11.7% vs. 18.4%), less often consulted general practices in the last three months (58.1% vs. 67.8%), and had less often inadequate health literacy (15.4% vs. 22.4%) (cf. Table 2b).

Patients advised to use specialist practices were younger (59 vs. 66 years), more often men (46.7% vs. 40.8%), had more often attained tertiary education (35.6% vs. 23.0%), better self-rated health (50 vs. 40 points) (cf. Table 2a), less often general and unspecified disorders (16.2% vs. 23.3%), more often health problems in the musculoskeletal system (28.4% vs. 21.9%), less often symptoms and complaints (62.7% vs. 68.7%), more often visited specialist practices (59.2% vs. 45.3%) and hospitals (28.2% vs. 21.7%) in the last three months, and had less often inadequate health literacy (11.6% vs. 22.4%) (cf. Table 2b).

Patients with recommendations for general practices had better self-rated health (50 vs. 40 points) (cf. Table 2a), less often symptoms and complaints (62.9% vs. 68.7%), and less often utilised emergency care in the last three months (12.6% vs. 18.1%) (cf. Table 2b). Patients who were offered to use telephone counselling were younger (61 vs. 66 years), more often women (65.5% vs. 59.0%), less often health problems in the musculoskeletal system (14.8% vs. 21.9%) and less often utilised emergency care in the last three months (13.3% vs. 18.1%) (cf. Table 2a). There were no larger differences between patients recommended to use emergency home visits and the total population (cf. Table 2a, b).

### Association between recommendations and patient characteristics

The associations between these data and the setting recommendations are shown in Table 3a–d. Recommendations for rescue service were associated with higher age (odds ratio for 20 years difference: 1.44, 95% confidence interval: 1.15/1.81, *p* = 0.002) and a higher subjective treatment urgency (odds ratio for 3 points difference: 2.51, 95% confidence interval: 1.83/3.43, *p* < 0.001) (cf. Table 3a). The setting emergency home visits was more often recommended if patients had worse self-rated health (odds ratio for 30 points difference: 0.67, 95% confidence interval: 0.55/0.81, *p* < 0.001) (cf. Table 3a).

**Table 3a:** Associations between sociodemographic and health-related data and setting allocations: results from multilevel, multivariable logistic regression models adjusted for random effects at the level of regions and months of observation within regions (n = 1201)

	Rescue service		Emergency home visit	
	OR (95% CI)	*p*	OR (95% CI)	*p*
***Age (per 20 years difference)***	***1.44 (1.15/1.81)***	***0.002***	1.17 (0.99/1.39)	0.062
Sex:				
- men or non-binary	reference		reference	
- women	0.90 (0.63/1.28)	0.550	0.98 (0.74/1.30)	0.907
Living arrangement:				
- living with others	reference		reference	
- living alone	1.02 (0.70/1.50)	0.909	1.04 (0.77/1.41)	0.810
Education (pursuant to CASMIN):				
- uncompleted, general elementary or basic vocational	reference		reference	
- secondary school certificate or “A” level equivalent	1.35 (0.90/2.03)	0.150	1.27 (0.90/1.80)	0.168
- higher or lower tertiary	0.85 (0.49/1.46)	0.550	1.26 (0.83/1.91)	0.279
Country of birth:				
- patient and both parents in Germany	reference		reference	
- patient in Germany and at least one parent abroad	1.54 (0.80/2.96)	0.201	0.93 (0.52/1.64)	0.798
- patient abroad	1.06 (0.57/1.97)	0.857	1.05 (0.63/1.74)	0.850
***Subjective treatment urgency *** ***(numerical rating scale; per 3 points difference)***	***2.51 (1.83/3.43)***	***< 0.001***	1.21 (0.97/1.52)	0.095
***Self-rated health *** ***(EQ-5D visual analogue scale; per 30 points difference)***	0.85 (0.67/1.09)	0.202	***0.67 (0.55/0.81)***	***< 0.001***
Depressiveness (pursuant to PHQ-2; per 3 points difference)	0.92 (0.68/1.26)	0.620	1.12 (0.87/1.45)	0.389
Health problem: organ system (pursuant to ICPC-2):				
- general and unspecified disorders - musculoskeletal system - digestive system - cardiovascular system - respiratory system - neurological system - urological system	1.49 (0.94/2.35) 1.03 (0.61/1.75) 1.21 (0.73/2.03) 2.18 (1.21/3.91) 1.20 (0.69/2.08) 1.25 (0.71/2.19) 0.72 (0.31/1.66)	0.087 0.910 0.463 0.009 0.525 0.446 0.436	1.46 (1.01/2.13) 1.43 (0.95/2.14) 0.82 (0.54/1.23) 0.69 (0.41/1.17) 0.86 (0.55/1.34) 1.41 (0.89/2.23) 1.25 (0.67/2.31)	0.046 0.084 0.334 0.167 0.510 0.140 0.488
Health problem: diagnosis type (pursuant to ICPC-2):				
- symptoms and complaints - infections - injuries - other diagnoses	0.69 (0.37/1.25) 0.67 (0.26/1.77) 1.25 (0.59/2.64) 0.92 (0.48/1.76)	0.220 0.424 0.563 0.808	1.05 (0.65/1.69) 1.75 (0.87/3.51) 0.57 (0.29/1.09) 1.06 (0.63/1.80)	0.844 0.115 0.091 0.822
Past health service use: number of contacts				
- with general practices - with specialist practices - with emergency care - with hospitals	1.08 (0.72/1.62) 0.61 (0.42/0.89) 1.58 (1.00/2.50) 1.16 (0.71/1.90)	0.723 0.010 0.050 0.557	1.00 (0.73/1.38) 1.00 (0.75/1.33) 0.72 (0.49/1.06) 1.69 (1.10/2.59)	0.983 0.984 0.097 0.016
Health literacy (pursuant to HLS-Q16-EU):				
- inadequate (0–8 points)	reference		reference	
- problematic (9–12 points)	0.99 (0.66/1.49)	0.977	0.76 (0.56/1.05)	0.092
- sufficient (13–16 points)	1.02 (0.64/1.65)	0.923	0.89 (0.60/1.31)	0.560

OR: odds ratio; 95% CI: 95% confidence interval; HLS- EU- Q16, European Health Literacy Questionnaire with 16 Items; ICPC-2, International Classification of PrimaryCare, Second Revision; PHQ-2, Patient Health Questionnaire 2

Recommendations for emergency departments were associated with better self-rated health (odds ratio for 30 points difference: 1.37, 95% confidence interval: 1.11/1.70, *p* = 0.003) and health problems classified as injuries (odds ratio: 3.12, 95% confidence interval: 1.67/5.83, *p* < 0.001) (cf. Table 3b). There was also a statistically significant association between recommendations for emergency practices and lower age (odds ratio for 20 years difference: 0.60, 95% confidence interval: 0.48/0.74, *p* < 0.001) (cf. Table 3b).

**Table 3b:** Associations between sociodemographic and health-related data and setting allocations: results from multilevel, multivariable logistic regression models adjusted for random effects at the level of regions and months of observation within regions (n = 1201) (continued)

	Emergency department		Emergency practice	
	OR (95% CI)	*p*	OR (95% CI)	*p*
***Age (per 20 years difference)***	1.05 (0.87/1.26)	0.635	***0.60 (0.48/0.74)***	***< 0.001***
Sex:				
- men or non-binary	reference		reference	
- women	1.01 (0.74/1.38)	0.956	1.11 (0.77/1.59)	0.576
Living arrangement:				
- living with others	reference		reference	
- living alone	1.02 (0.73/1.42)	0.918	0.89 (0.59/1.34)	0.581
Education (pursuant to CASMIN):				
- uncompleted, general elementary or basic vocational	reference		reference	
- secondary school certificate or “A” level equivalent - higher or lower tertiary	1.10 (0.76/1.61) 0.91 (0.57/1.45)	0.611 0.698	1.37 (0.83/2.26) 1.13 (0.64/1.99)	0.213 0.672
Country of birth:				
- patient and both parents in Germany	reference		reference	
- patient in Germany and at least one parent abroad - patient abroad	0.75 (0.39/1.43) 0.65 (0.35/1.19)	0.381 0.163	0.82 (0.42/1.63) 0.58 (0.28/1.19)	0.579 0.138
Subjective treatment urgency (numerical rating scale; per 3 points difference)	1.35 (1.06/1.73)	0.015	1.10 (0.84/1.45)	0.495
***Self-rated health *** ***(EQ-5D visual analogue scale; per 30 points difference)***	***1.37 (1.11/1.70)***	***0.003***	1.15 (0.89/1.47)	0.278
Depressiveness (pursuant to PHQ-2; per 3 points difference)	1.11 (0.84/1.46)	0.476	0.96 (0.68/1.36)	0.823
Health problem: organ system (pursuant to ICPC-2):				
- general and unspecified disorders - musculoskeletal system - digestive system - cardiovascular system - respiratory system - neurological system - urological system	0.65 (0.42/0.98) 1.05 (0.67/1.64) 1.73 (1.11/2.72) 1.03 (0.58/1.83) 1.09 (0.66/1.79) 0.72 (0.42/1.25) 1.32 (0.68/2.58)	0.042 0.846 0.017 0.913 0.736 0.246 0.409	0.66 (0.40/1.08) 1.21 (0.73/2.03) 0.50 (0.27/0.93) 1.02 (0.50/2.08) 1.29 (0.75/2.23) 0.98 (0.53/1.82) 1.16 (0.52/2.59)	0.096 0.460 0.029 0.964 0.361 0.950 0.709
***Health problem: diagnosis type (pursuant to ICPC-2)***:				
- symptoms and complaints - infections ***- injuries*** - other diagnoses	1.84 (1.10/3.07) 1.12 (0.51/2.50) ***3.12 (1.67/5.83)*** 1.59 (0.90/2.82)	0.020 0.775 ***< 0.001*** 0.114	1.69 (0.94/3.01) 1.88 (0.82/4.29) 1.58 (0.75/3.34) 0.89 (0.45/1.77)	0.077 0.135 0.230 0.738
Past health service use: number of contacts				
- with general practices - with specialist practices - with emergency care - with hospitals	1.03 (0.72/1.46) 0.88 (0.64/1.22) 1.40 (0.93/2.13) 1.33 (0.86/2.06)	0.874 0.449 0.111 0.203	0.85 (0.57/1.25) 1.21 (0.83/1.77) 0.78 (0.45/1.38) 1.74 (1.00/3.03)	0.406 0.324 0.396 0.052
Health literacy (pursuant to HLS-Q16-EU):				
- inadequate (0–8 points)	reference		reference	
- problematic (9–12 points)	1.18 (0.83/1.67)	0.358	0.91 (0.62/1.34)	0.643
- sufficient (13–16 points)	1.26 (0.82/1.94)	0.289	0.63 (0.36/1.09)	0.098

OR: odds ratio; 95% CI: 95% confidence interval; HLS- EU- Q16, European Health Literacy Questionnaire with 16 Items; ICPC-2, International Classification of PrimaryCare, Second Revision; PHQ-2, Patient Health Questionnaire 2

Recommendations for specialist practices were associated with health problems classified as symptoms or complaints (odds ratio: 3.75, 95% confidence interval: 1.49/9.45, *p* = 0.005) (cf. Table 3c). The setting “general practices” was more often recommended if patients had lower subjective treatment urgency (odds ratio for 3 points difference: 0.58, 95% confidence interval: 0.44/0.76, *p* < 0.001) and health problems not classified as injuries (odds ratio: 0.26, 95% confidence interval: 0.10/0.68, *p* = 0.006) (cf. Table 3c).

**Table 3c:** Associations between sociodemographic and health-related data and setting allocations: results from multilevel, multivariable logistic regression models adjusted for random effects at the level of regions and months of observation within regions (n = 1201) (continued)

	Specialist practice		General practice	
	OR (95% CI)	*p*	OR (95% CI)	*p*
***Age (per 20 years difference)***	0.82 (0.59/1.14)	0.231	0.95 (0.76/1.18)	0.630
Sex:				
- men or non-binary	reference		reference	
- women	1.17 (0.66/2.08)	0.592	1.25 (0.86/1.80)	0.239
Living arrangement:				
- living with others	reference		reference	
- living alone	1.17 (0.63/2.19)	0.619	1.36 (0.92/2.01)	0.129
Education (pursuant to CASMIN):				
- uncompleted, general elementary or basic vocational	reference		reference	
- secondary school certificate or “A” level equivalent - higher or lower tertiary	1.48 (0.63/3.49) 2.45 (0.99/6.09)	0.367 0.053	0.70 (0.44/1.11) 0.52 (0.30/0.90)	0.127 0.019
Country of birth:				
- patient and both parents in Germany	reference		reference	
- patient in Germany and at least one parent abroad - patient abroad	1.46 (0.57/3.75) 0.34 (0.08/1.51)	0.436 0.155	1.56 (0.82/2.95) 0.96 (0.47/1.96)	0.176 0.905
***Subjective treatment urgency *** ***(numerical rating scale; per 3 points difference)***	0.65 (0.42/0.99)	0.043	***0.58 (0.44/0.76)***	***< 0.001***
Self-rated health (EQ-5D visual analogue scale; per 30 points difference)	1.34 (0.90/2.01)	0.153	1.06 (0.82/1.38)	0.643
Depressiveness (pursuant to PHQ-2; per 3 points difference)	1.77 (1.08/2.89)	0.023	1.02 (0.73/1.44)	0.903
Health problem: organ system (pursuant to ICPC-2):				
- general and unspecified disorders - musculoskeletal system - digestive system - cardiovascular system - respiratory system - neurological system - urological system	0.49 (0.21/1.17) 0.83 (0.38/1.82) 0.49 (0.18/1.30) 0.62 (0.20/1.87) 0.25 (0.07/0.91) 0.33 (0.09/1.17) 0.40 (0.08/1.98)	0.109 0.644 0.149 0.394 0.036 0.087 0.261	1.68 (1.03/2.76) 1.10 (0.63/1.92) 1.05 (0.59/1.87) 0.53 (0.25/1.12) 0.86 (0.47/1.56) 1.01 (0.53/1.92) 0.83 (0.31/2.17)	0.038 0.736 0.860 0.097 0.611 0.972 0.698
***Health problem: diagnosis type (pursuant to ICPC-2)***:				
***- symptoms and complaints*** - infections ***- injuries*** - other diagnoses	***3.75 (1.49/9.45)*** 0.96 (0.11/8.30) 2.40 (0.77/7.47) 1.92 (0.69/5.39)	***0.005*** 0.972 0.131 0.214	0.55 (0.30/1.03) 0.67 (0.24/1.88) ***0.26 (0.10/0.68)*** 1.25 (0.63/2.46)	0.061 0.446 ***0.006*** 0.522
Past health service use: number of contacts				
- with general practices - with specialist practices - with emergency care - with hospitals	0.86 (0.46/1.62) 1.66 (0.91/3.05) 2.15 (0.97/4.73) 0.62 (0.24/1.61)	0.647 0.099 0.058 0.324	1.28 (0.84/1.94) 0.95 (0.65/1.39) 1.12 (0.65/1.92) 0.47 (0.25/0.92)	0.244 0.792 0.686 0.027
Health literacy (pursuant to HLS-Q16-EU):				
- inadequate (0–8 points)	reference		reference	
- problematic (9–12 points)	1.08 (0.58/2.00)	0.815	1.02 (0.68/1.54)	0.911
- sufficient (13–16 points)	0.56 (0.21/1.46)	0.233	0.92 (0.54/1.57)	0.753

OR: odds ratio; 95% CI: 95% confidence interval; HLS- EU- Q16, European Health Literacy Questionnaire with 16 Items; ICPC-2, International Classification of PrimaryCare, Second Revision; PHQ-2, Patient Health Questionnaire 2

Finally, there was a statistically significant association between the recommended setting “telephone counselling” and lower age (odds ratio for 20 years difference: 0.71, 95% confidence interval: 0.59/0.85, *p* < 0.001), lower subjective treatment urgency (odds ratio for 3 points difference: 0.65, 95% confidence interval: 0.51/0.82, *p* < 0.001) and health problems not classified as symptoms and complaints (odds ratio: 0.41, 95% confidence interval: 0.25/0.68, *p* = 0.001) or infections (odds ratio: 0.22, 95% confidence interval: 0.09/0.57, *p* = 0.002) (cf. Table 3d).

**Table 3d:** Associations between sociodemographic and health-related data and setting allocations: results from multilevel, multivariable logistic regression models adjusted for random effects at the level of regions and months of observation within regions (n = 1201) (continued)

	Telephone counselling	
	OR (95% CI)	*p*
***Age (per 20 years difference)***	***0.71 (0.59/0.85)***	***< 0.001***
Sex:		
- men or non-binary	reference	
- women	0.81 (0.59/1.11)	0.184
Living arrangement:		
- living with others	reference	
- living alone	1.19 (0.85/1.66)	0.314
Education (pursuant to CASMIN):		
- uncompleted, general elementary or basic vocational	reference	
- secondary school certificate or “A” level equivalent - higher or lower tertiary	1.21 (0.81/1.79) 1.11 (0.70/1.78)	0.354 0.655
Country of birth:		
- patient and both parents in Germany	reference	
- patient in Germany and at least one parent abroad - patient abroad	1.23 (0.70/2.16) 1.42 (0.84/2.39)	0.470 0.191
***Subjective treatment urgency *** ***(numerical rating scale; per 3 points difference)***	***0.65 (0.51/0.82)***	***< 0.001***
Self-rated health (EQ-5D visual analogue scale; per 30 points difference)	1.03 (0.83/1.28)	0.797
Depressiveness (pursuant to PHQ-2; per 3 points difference)	0.98 (0.74/1.30)	0.900
Health problem: organ system (pursuant to ICPC-2):		
- general and unspecified disorders - musculoskeletal system - digestive system - cardiovascular system - respiratory system - neurological system - urological system	1.25 (0.83/1.88) 0.79 (0.49/1.27) 1.26 (0.79/2.01) 1.76 (1.01/3.05) 1.04 (0.63/1.70) 1.17 (0.72/1.93) 1.32 (0.62/2.78)	0.286 0.324 0.335 0.046 0.883 0.526 0.473
***Health problem: diagnosis type (pursuant to ICPC-2)***:		
***- symptoms and complaints*** ***- infections*** - injuries - other diagnoses	***0.41 (0.25/0.68)*** ***0.22 (0.09/0.57)*** 0.40 (0.20/0.81) 0.66 (0.38/1.15)	***0.001*** ***0.002*** 0.010 0.145
Past health service use: number of contacts		
- with general practices - with specialist practices - with emergency care - with hospitals	0.94 (0.67/1.32) 1.28 (0.93/1.76) 1.51 (0.98/2.32) 0.63 (0.39/1.03)	0.718 0.133 0.063 0.064
Health literacy (pursuant to HLS-Q16-EU):		
- inadequate (0–8 points)	reference	
- problematic (9–12 points)	1.25 (0.89/1.76)	0.202
- sufficient (13–16 points)	1.44 (0.93/2.22)	0.101

OR: odds ratio; 95% CI: 95% confidence interval; HLS- EU- Q16, European Health Literacy Questionnaire with 16 Items; ICPC-2, International Classification of PrimaryCare, Second Revision; PHQ-2, Patient Health Questionnaire 2

There were no statistically significant associations between setting recommendations and other sociodemographic data (including sex) or indicators of health status, healthcare utilisation or health literacy.

## Discussion

The aim of our study was to identify associations between patient characteristics and recommendations for the different treatment settings. Most findings indicate that the settings are frequently recommended due to their specific function within the healthcare system. For example, patients with higher treatment urgency were visited by the rescue service, patients with low self-rated health received home visits by outpatient physicians, patients with health problems classified as symptoms or complaints were sent to outpatient specialist care and patients with low treatment urgency should visit general practice. Nevertheless, patients with better self-rated health had a higher likelihood of being advised to visit the emergency department, which might point to recommendations based on patient preferences such as availability of medical imaging rather than medical needs. However, self-rated health might not always agree with objective health assessments such as SmED. And certain health problems regularly treated in emergency departments such as injuries, might also go along with (relatively) good self-rated health. In this context it also needs to be mentioned that our study does not inform about the appropriateness of given recommendations.

There were other studies on the appropriateness of computer-assisted initial assessments. SMASS was analysed in a prospective surveillance study in 2012. Decisions of nurses assisted by a computerised decision support system were compared to retrospective assessments of hospital physicians and primary care physicians. There was low agreement between the assessments of the three groups. Seven of the 153 examined cases appeared to be under-triaged and a risk to health or life was identified in one case. The authors concluded that computer-assisted telephone triage was safe, but required competent specialists with dedicated training in communication [28]. In a recent study, which included 2543 patients and analysed the current version of SMASS, no cases of potentially hazardous undertriage were found [15]. An evaluation of nurse telephone consultations aided by a computer-based call management system in 1998 reported that overall workload of general practitioners was reduced by half and faster access to advice was facilitated. Moreover, there was no increase in the number of adverse events [29]. A 2007 study from Sweden on computer-supported telephone nurse triage reported that 97.6% of 362 participants were referred to the appropriate level of care [30].

Only a small number of studies took the associations between patient characteristics and recommendations by medical on-call services into account. For example, similar to our results, older age was also associated with higher rates of referral for home visits in a nursed-led out-of-hours telephone triage and advice service in general practice [8]. In another study, older adults and patients with diabetes mellitus, dementia, or previous cerebral infarction were at risk of undertriage [9]. Moreover, individuals living in rural areas had fundamentally different pattern of service use than individuals living in urban areas. Rural callers were more likely to use family physicians and less likely to use emergency departments after being advised to use the respective service [31]. Nakubulwa et al. described a better compliance in following the advice given by a non-emergency medical helpline for children aged less than 16 years, women, and individuals of Asian/Asian British ethnicity [32].

### Strengths and limitations

Our study has a comparably low participation rate of 18.5% which could be a consequence of using contact data of patients, which were used for healthcare and not for conducting studies. Many exclusion criteria could not be assessed systematically. It is possible that some patients, who had been contacted, had died without our knowledge or could not respond because of bad health condition or functional limitations. It is also possible that some patients gave wrong contact data. If patients lived in a hotel or holiday apartment or were at a friend’s place while utilising the telephone services they could possibly not be reached for our study. Nevertheless, a low participation rate may also reflect unwillingness to participate in the study.

In a non-responder analysis, the proportion of women (58.6%) and men (41.2%) in our 2020 data set were comparable to the official numbers in the SmED evaluation report from 2020 (58.3%, and 41.7%, respectively) [19]. Unfortunately, due to the inclusion of minors in the report, differences in the age group below 50 years could not be compared. Among the older patients, the age group 50–65 was comparable between study and report (31.2% vs. 33.5%), but patients aged 66–80 years were overrepresented (41.9% vs. 35.2%) and over 80 years old patients were underrepresented in our study (26.9% vs. 31.3%). In addition to this age-based selection bias, there might be other factors like health status or educational level that biased the selection of participants in our study, but these factors could not be analysed in our non-responder analysis.

No sample size calculation was conducted, therefore statistical power to detect associations might be lower than usual. With regard to the statistical analyses it needs to be mentioned that the likelihood of type-II errors is generally increased by the use of Bonferroni-corrections [33]. The risk for undetected associations is highest in the setting “specialist care”, which only comprised 75 patients. In each of the other settings, the size of the subsample exceeds 200 patients.

The association between patient characteristics and recommendations by computer-assisted initial assessments is probably to a certain degree associated with the specific algorithms in such a tool. Therefore, the results might not be fully generalizable on other digital tools. Additionally, differences in healthcare systems and cultural values between different countries might further affect generalisability of our results. It also needs to be mentioned that 17.1% of the initial assessments were made for COVID-19-related consultation reasons, which might have influenced the recommended settings. For example, most patients fearing to be infected with COVID-19 have probably received telephone counselling, which therefore might be overrepresented in our study.

We used validated and established instruments like PHQ-2 [23] and HLS-Q16-EU [26]. The answers of the patients might have been affected by recall bias due to the fact that the questionnaire was sent out between 4 and 72 days after receiving the intervention. Also errors and social desirability might have affected the data quality. In contrast to these limitations, it is worth emphasizing that the large sample size and multilevel, multivariable methods allowed a detailed analysis.

## Conclusions

The DEMAND intervention aimed to improve patient allocation in emergency care by computer-assisted structured initial assessment. Most associations between specific recommendations and patient characteristics were comprehensible and in line with the aim of the intervention. For example, patients with higher treatment urgency more often received recommendations for the rescue service and patients with lower treatment urgency recommendations for primary care. However, it should be clarified why patients with better self-rated health were more likely to receive recommendations for emergency departments.

## Data Availability

De-identified data are available upon reasonable request from the corresponding author.
